# Incomplete Information Management Using an Improved Belief Entropy in Dempster-Shafer Evidence Theory

**DOI:** 10.3390/e22090993

**Published:** 2020-09-07

**Authors:** Bin Yang, Dingyi Gan, Yongchuan Tang, Yan Lei

**Affiliations:** School of Big Data and Software Engineering, Chongqing University, Chongqing 401331, China; 20181665@cqu.edu.cn (B.Y.); 20181668@cqu.edu.cn (D.G.); yanlei@cqu.edu.cn (Y.L.)

**Keywords:** Dempster–Shafer evidence theory, belief entropy, Deng entropy, uncertainty management, incomplete information fusion

## Abstract

Quantifying uncertainty is a hot topic for uncertain information processing in the framework of evidence theory, but there is limited research on belief entropy in the open world assumption. In this paper, an uncertainty measurement method that is based on Deng entropy, named Open Deng entropy (ODE), is proposed. In the open world assumption, the frame of discernment (FOD) may be incomplete, and ODE can reasonably and effectively quantify uncertain incomplete information. On the basis of Deng entropy, the ODE adopts the mass value of the empty set, the cardinality of FOD, and the natural constant *e* to construct a new uncertainty factor for modeling the uncertainty in the FOD. Numerical example shows that, in the closed world assumption, ODE can be degenerated to Deng entropy. An ODE-based information fusion method for sensor data fusion is proposed in uncertain environments. By applying it to the sensor data fusion experiment, the rationality and effectiveness of ODE and its application in uncertain information fusion are verified.

## 1. Introduction

Uncertain information processing is applied to complex systems in many fields, such as sensor networks [1,2], pattern recognition [3,4], and supply chain network management [5,6]. Dempster–Shafer (D–S) evidence theory [7,8,9] has a good performance in dealing with uncertain information, such as reliability assessment [10,11], pattern recognition [12,13], decision-making [14,15,16], and so on [17,18,19,20]. The sources of uncertainty information of D-S evidence theory include: (1) mass function of focal element, (2) non-zero mass function of empty set, and (3) uncertain information represented by possible incomplete FOD. Previously, many scholars have proposed many uncertainty measurement methods, which can identify the difference of uncertain information in probability framework to different degrees and are widely used in different fields. As the most widely used information entropy theory, Shannon entropy has been extended to a variety of fields, such as network entropy in complex networks [21] and gene enlargement analysis in the field of biological information [22]. Uncertainty measure for uncertain information management is a hot topic [23,24,25,26,27]. Entropy-based measure attracts lots of attention among researches [28,29]. Inspired by Shannon entropy, in the framework of evidence theory, many scholars have proposed methods in order to measure the uncertainty of evidence from different perspectives, such as Yager’s dissonance measure [30], Deng entropy [31,32], and so on [17,33]. Meanwhile, some improved belief entropy based on Deng entropy has been proposed by scholars, such as Zhou et al.’s entropy [34] and Cui et al.’s entropy [35]. Belief entropy has shown its advantages for addressing uncertain information processing in some practical applications, such as risk analysis [36,37,38], decision-making [15,33] and sensor data fusion [39]. According to the introduction to belief entropy in [40], the existing methods are mainly for complete information in the closed world conditions.

In the open world hypothesis, the empty set of non-zero mass functions means that the FOD is incomplete. The incomplete uncertain information is modeled by non-zero mass function of empty set, so we can use the empty set of non-zero mass functions in order to model the incomplete information in the open world. For this reason, the reasonable measure should take into consideration of the incomplete information that was caused by the empty set of non-zero mass functions, and at the same time, the uncertainty of the FOD should also be considered [41]. Thus, uncertainty measure can be applied in the open world assumption [42]. An improved belief entropy named Open Deng entropy (ODE) is proposed in this work in order to solve the uncertainty measurement of open world assumption. The ODE takes into account the sources of uncertain information in the Dempster–Shafer evidence theory framework that are rarely considered by other existing methods, including the uncertain information brought by the incomplete FOD and the non-zero mass function of empty set. This article gives some examples and the application of ODE in sensor data fusion to further discuss and verify the validity and applicability of the ODE. First, BPA is adopted to model the uncertain information, then, ODE is used to measure the uncertainty of BPA. After that, calculating the weight of each evidence function for modifying the mass function. Finally, data fusion using Dempster’s combination rule and applying the method to engineering applications.

The remainder of this article is shown below. Section 2 covers the preliminary knowledge. In Section 3, the ODE is proposed, and its properties are discussed, and some numerical examples are given. In Section 4, the information fusion method that is based on ODE and its application in sensor data are introduced. Finally, the conclusion of this paper is drawn in Section 5.

## 2. Preliminaries

This section provides a brief overview of some preliminaries.

### 2.1. Dempster-Shafer Evidence Theory

**Definition** **1.** *The frame of discernment *Ω* is defined as a finite non-empty set containing N mutually exclusive events, and its specific expression is as follows:*

(1) $$\Omega =\left\{\Theta_{1},\Theta_{2},\ldots ,\Theta_{i},\ldots ,\Theta_{N}\right\}.$$

**Definition** **2.** *The power set of *Ω*, denoted as $2^{\Omega }$, which is composed of $2^{N}$ elements, is defined, as follows:*

(2) $$2^{\Omega }=\left\{\phi ,\left\{\Theta_{1}\right\},\left\{\Theta_{2}\right\},\ldots ,\left\{\Theta_{N}\right\},\left\{\Theta_{1},\Theta_{2}\right\},\ldots ,\left\{\Theta_{1},\Theta_{2},\Theta_{i}\right\},\ldots ,\Omega \right\}.$$

**Definition** **3.** *For *Ω*, a basic probability assignment(BPA)(or mass function) is a mapping m: $2^{\Omega }\to \left[0,1\right]$, which satisfies the following properties:*

(3) $$m\left(\phi \right)=0,\sum_{A\in \Omega }^{}m\left(A\right)=1.$$

*If m(A)>0, the subset A is called a focal element and m(A)>0 is the mass function value of proposition subset A.*

**Definition** **4.** *A body of evidence (BOE) is a component unit of uncertain information based on FOD, power set of FOD, and mass function. A BOE is a binary group of proposition subset and corresponding mass function, which is defined as:*

(4) $$\left(ℜ,m\right)=\left\{<A,m\left(A\right)>:A\in 2^{\Omega },m\left(A\right)>0\right\},$$

*where ℜ is a subset of the power set $2^{\Omega }$.*

**Definition** **5.** *For *Ω*, the belief function Bel or the plausibility function Pl, is defined as*

(5) $$Bel\left(A\right)=\sum_{\phi \neq B\subseteq A}^{}m\left(B\right),$$

(6) $$Pl\left(A\right)=\sum_{B\cap A\neq \phi }^{}m\left(B\right).$$

**Definition** **6.** *In Dempster–Shafer (D–S) evidence theory, Dempster’s rule of combination can fuse two independent mass functions $m_{1}$ and $m_{2}$:*

(7) $$m\left(A\right)=\left(m_{1}⨁m_{2}\right)\left(A\right)=\frac{1}{1-K}\sum_{B\cap C=A}^{}m_{1}\left(B\right)m_{2}\left(C\right),$$

*where k is a normalization factor defined, as follows:*

(8) $$k=\sum_{B\cap C=\phi }^{}m_{1}\left(B\right)m_{2}\left(C\right).$$

*It is worth noting that the classical definitions of DST are defined and used in the closed world.*

**Definition** **7.** *In the open world hypothesis, Dempster’s rule of combination is extended by Deng in [31]. The intersection of empty set and empty set is still empty set, which satisfies condition $\phi_{1}\cap \phi_{2}=\phi$. Given two BPAs ($m_{1}$ and $m_{2}$), the generalized combination rule is defined, as follows:*

(9) $$m\left(A\right)=\frac{\left(1-m\left(\phi \right)\right)\sum_{B\cap C=A}m_{1}\left(B\right)\cdot m_{2}\left(C\right)}{1-K},$$

(10) $$K=\sum_{B\cap C=\phi }^{}m_{1}\left(B\right)m_{2}\left(C\right),$$

(11) $$\begin{array}{ccc} m(\phi ) & = & m_{1}\left(\phi \right)\cdot m_{2}\left(\phi \right), \end{array}$$

(12) $$\begin{array}{ccc} m(\phi ) & = & 1\;if\;\;and\;\;only\;\;if\;K=1. \end{array}$$

### 2.2. Shannon Entropy and Belief Entropy

**Definition** **8.** *As the most widely used information entropy theory, Shannon entropy has been extended to many fields, such as network entropy in complex networks [21] and gene enlargement analysis in the field of biological information [22]. Shannon entropy is defined as [43]:*

(13) $$H=-\sum_{i=1}^{N}p_{i}log_{b}p_{i},$$

*where N is the number of basic states, $p_{i}$ is the probability of state i, and $p_{i}$ satisfied $\sum_{i=1}^{N}p_{i}=1$.*

**Definition** **9.** *Deng entropy was proposed in [31] based on Shannon entropy. Some properties and behaviors are discussed in [31,44]. Deng entropy is defined as [31]:*

(14) $$E_{d}\left(m\right)=-\sum_{A\subseteq X}^{}m\left(A\right)log_{2}\frac{m(A)}{2^{\left|A\right|}-1},$$

*where |A| represents the cardinality of the proposition A. According to [31], there are some advantages in Deng entropy when compared with other methods. However, Deng entropy also has some significant disadvantages. For example, Deng entropy does not take into account the influence of the size of the FOD and the intersection of different proposition subsets [35], and it cannot be applied to incomplete FOD.*

**Definition** **10.** *As an improvement on Deng entropy, Cui et al.’s entropy was proposed in [35]. When compared with Deng entropy, the improved entropy in [35] takes the influence of the size of the FOD and the intersection of different proposition subsets into account. Cui et al.’s entropy is defined as [35]:*

(15) $$E_{Cui}\left(m\right)=-\sum_{A\subseteq X}^{}m\left(A\right){log_{2}}^{}\left(\frac{m(A)}{2^{\left|A\right|}-1}e^{\sum_{B\subseteq X\wedge B\neq A}^{}\frac{\left|A\cap B\right|}{2^{\left|X\right|}-1}}\right),$$

*where |A| represents the cardinality of the proposition A, X is the frame of discernment, |X| denotes the certain element number in the frame of discernment, and $\left|A\cap B\right|$ is the cardinality of the intersection of A and B. Although Cui et al.’s entropy is optimized for Deng entropy, according to [45], Cui et al.’s entropy still has some obvious problems, such as its lack of subadditivity, additivity, and monotonicity.*

## 3. The Improved Belief Entropy

In this part, we define a new belief entropy for incomplete and uncertain information measuring based on Deng entropy named Open Deng entropy (ODE). On this basis, a sensor data fusion method is proposed and its advantages as compared with other methods are discussed in Section 4.

### 3.1. The Open Deng Entropy

The Open Deng entropy (ODE) is defined as follows:

(16) $$E_{ode}=-\sum_{\left(A\subseteq X)\wedge (A\neq \phi \right)}^{}m\left(A\right)log_{2}\left(\frac{m(A)}{2^{\left|A\right|}-1}e^{\frac{m(\phi )}{\left|X\right|}}\right),$$

where *X* is the frame of discernment, |X| represents the number of elements identified in the FOD, |A| denotes the element number in proposition *A*, the open world characteristic factor of D–S evidence theory is a newly proposed parameter. Through the open world characteristic factor, the ODE can include the non-zero mass function of the empty set and the uncertain information expressed by the possible imperfection of the FOD. The basis of the open world characteristic factor is as follows.

- The parameter m(ϕ) represents the value of mass function of empty set, and parameter $\left|X\right|$ is the potential of the FOD. In the evidence theoretical framework, the two have clear physical meanings.
- When the information space degenerates from the open world to the closed world, the value of the empty set mass function is 0, which keeps good compatibility with the improved Deng entropy method.

**Property** **1.** *semantic consistency with evidence theory. ODE is defined based on mass function, the frame of discernment and its potential in the evidence theory, propositional subset of the FOD, and its potential and empty set, which does not involve the loss of semantic consistency with the evidence theory caused by mass function and probability function conversion in the closed world described in literature [40], so the ODE satisfies the semantic consistency with the evidence theory.*

**Property** **2.** *non-negative. In the open world, the focal element proposition A defined by the classical evidence theory can also represent the empty set with incomplete information propositions in the open world, and its corresponding mass function value satisfies 1>m(A)>0. If and only if m(A)=1, which is, when the mass function is the Bayes probability, $E_{ode}\left(m\right)=0$. Regardless of whether the value of the BPA of the empty set is zero, which is, whether it is in the open world or the closed world, $\frac{m(A)}{2^{\left|A\right|}-1}e^{\frac{m(\phi )}{\left|X\right|}}$ is always not greater than 1. That is, $log_{2}\left(\frac{m(A)}{2^{\left|A\right|}-1}e^{\frac{m(\phi )}{\left|X\right|}}\right)$ is never greater than 0. Therefore, the Open Deng entropy satisfies the value non-negative characteristic.*

**Property** **3.** *probabilistic consistency. When proposition A is a subset of a single element, the ODE is reduced to $E_{d}\left(m\right)=-\sum_{A\subseteq X}^{}m\left(A\right)log_{2}\frac{m(A)}{2^{\left|A\right|}-1}$, which is fully compatible with the characteristic of Thomas Bayes probabilistic information of Shannon entropy measurement and has probability consistency. When proposition A is the empty set representing incomplete information under the assumption of open world, the confidence uncertainty of the mass function of the empty set and the size of the FOD affect the expression of probability consistency.*

The above three properties are completely consistent with Deng entropy. ODE and the improved entropy in [35] are both improved based on Deng entropy, but there are some differences between them. Cui et al.’s entropy is an improvement of Deng entropy when considering the influence of the size of the FOD and the intersection of different proposition subsets [35]. In contrast, the ODE improves Deng entropy by taking into account not only the size of the FOD, but also the non-zero mass function of empty set, so that the ODE can be applied to incomplete FOD, which shown in Example 3 and Section 4. It is important to note that the ODE, like Deng entropy and Cui et al.’s entropy, does not satisfy additivity, monotonicity, etc. With the addition of the non-zero mass function of empty set and incomplete FOD, information uncertainty in the open world becomes more complex. Whether ODE needs to satisfy the additivity, monotonicity, and other properties of closed world confidence entropy proposed by researchers requires further study by more scholars. At the same time, inspired by Cui et al.’s entropy, the influence of the intersection of different proposition subsets can be taken into account in the future development of ODE.

### 3.2. Numerical Example and Discussion

**Example** **1.** *In the frame of discernment $X=\left\{a\right\}$, the BPAS are as follows:*

(17) m(a)=1.0,m(ϕ)=0.

In this example, the mass function of the empty set is 0, which shows that the mass function is distributed in the closed world. Shannon entropy *H*, Deng entropy $E_{d}$, and ODE $E_{ode}$ are calculated, as follows:

(18) $$H\left(m\right)=0,E_{d}\left(m\right)=0,E_{ode}\left(m\right)=0.$$

**Example** **2.** *In the frame of discernment $X=\left\{a,b,c,d\right\}$, the given mass function is as follows:*

(19) $$m\left(\left\{a\right\}\right)=0.25,\left(\left\{b\right\}\right)=0.25,m\left(\left\{c\right\}\right)=0.25,m\left(\left\{d\right\}\right)=0.25,m\left(\phi \right)=0.$$

In this example, the mass function of the empty set is 0, which indicates that the mass function is distributed in the closed world. Shannon entropy *H*, Deng entropy $E_{d}$, and ODE $E_{ode}$ are calculated, as follows:

(20) $$H\left(m\right)=2.0,E_{d}\left(m\right)=2.0,E_{ode}\left(m\right)=2.0.$$

From the measurement results of evidence uncertainty in the above two examples, it can be found that the mass function of the empty set is 0, which is, under the condition of the closed world, the ODE is reduced to the Deng entropy, and the calculated results are consistent with the results of the Deng entropy and Shannon entropy measurements. However, the mass function of the empty set in the open world is not zero, so Shannon entropy and Deng entropy are no longer applicable. At this time, we can only adopt the method of this chapter.

**Example** **3.** *In a changing FOD |X|, the given mass function is as follows:*

(21) $$m\left(\left\{a\right\}\right)=0.2,m\left(\left\{b\right\}\right)=0.3,m\left(\phi \right)=0.5.$$

**Figure 1:**
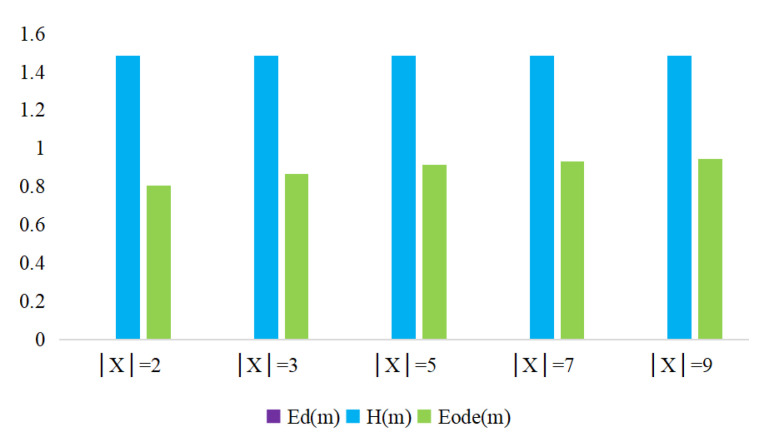
Uncertainty measuring results in Example 3.

The BPA of an empty set is 0.5, which is non-zero, which indicates that the mass function is allocated under open world conditions. The uncertainty measurement results of Shannon entropy *H*, Deng entropy $E_{d}$ and Open Deng entropy $E_{ode}$ varied with the changing FOD |X|, as shown in Table 1 and Figure 1. The measurement results show that, even if we regard the empty set proposition as a special uncertainty proposition with non-zero mass function assignment, Shannon entropy *H* can be used to calculate this example, the measurement results cannot reflect the change of potential in the FOD. Because the BPA of the empty set is non-zero, the Deng entropy cannot be applied in this example. Obviously, the Open Deng entropy can be used to $E_{ode}\left(m\right)$ indicate that, as the FOD |X| expands, the value of the measurement of evidence uncertainty gradually increases.

## 4. Application in Sensor Data Fusion with Incomplete Information

**Figure 2:**
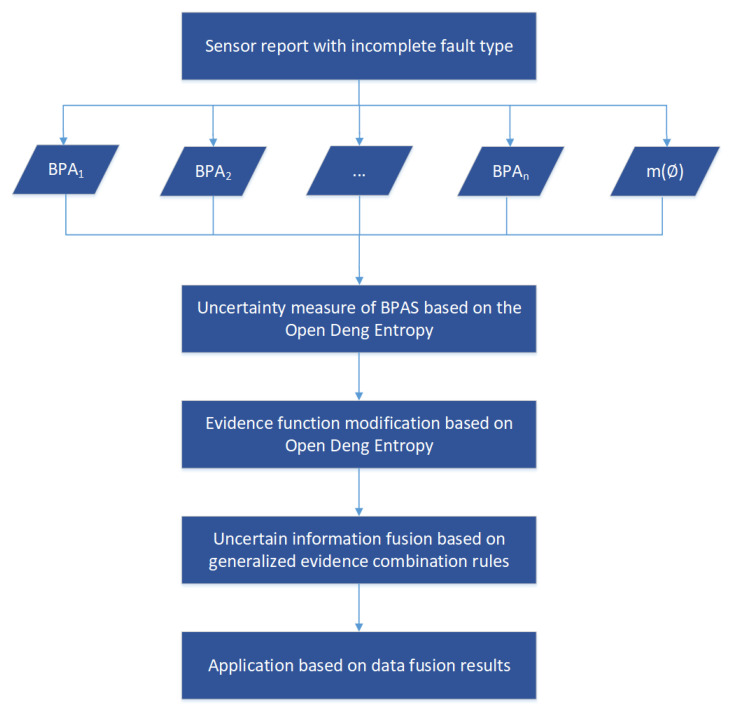
Sensor data fusion framework based on ODE.

This section proposes a conflict data fusion method that is based on the uncertainty of Open Deng entropy measurement information to illustrate the applicability and effectiveness of Open Deng entropy in information fusion. Figure 2 designs the framework of open world uncertain information fusion method based on Open Deng entropy. Detailed process description of steps in Figure 2 are as follows.

Step 1: in the open world, there is a lot of uncertain information in the practical application of BPA modeling. To address the uncertain information systematically and objectively, in the framework of Dempster–Shafer evidence theory, the first step is to use BPA to model the uncertain information.

Step 2: use Open Deng entropy to measure the uncertain information of BPA before further processing the data, it is necessary to use a reasonable and applicable uncertainty measure to measure the uncertainty of the information modeled by BPA in step 1. In this method, the uncertain information is measured by the Open Deng entropy. The uncertainty that corresponds to ODE is calculated, as follows:

(22) $$E_{ode}=-\sum_{\left(A\subseteq X)\wedge (A\neq \phi \right)}^{}m\left(A\right)log_{2}\left(\frac{m(A)}{2^{\left|A\right|}-1}e^{\frac{m(\phi )}{\left|X\right|}}\right)$$

Step 3: calculate the weight of each evidence function and modify the mass function based on the weight. The weight of each evidence function can be calculated according to the value of the ODE. The specific formula is as follows:

(23) $$w_{i}=\frac{E_{ode}\left(m_{i}\right)}{\sum_{i=1}^{n}E_{ode}\left(m_{i}\right)}$$

As the data preprocessing before evidence fusion, the weighted mass function of each proposition should be calculated by weight. The weight mass function mass is calculated, as follows:

(24) $$m_{w}\left(A\right)=\sum_{i=1}^{n}w_{i}m_{i}\left(A\right)$$

Step 4: data fusion using Dempster’s combination rule. This method use Open Deng entropy to transform and measure the conflict between different evidences, and the combination rule of Dempster is used to complete the data fusion. The combination result of each proposition *A* can be obtained through (n−1) Dempster’s combination rule:

(25) $$m\left(A\right)={\left({\left({\left({\left(m_{w}⨁m_{w}\right)}_{1}⨁m_{w}\right)}_{2}\ldots ⨁m_{w}\right)}_{n-2}⨁m_{w}\right)}_{n-1}\left(A\right),n\geq 2$$

Step 5: apply the method to engineering applications that need decision analysis.

The fault diagnosis experiment of motor rotor in literature [41] is taken as an application example, and its fault characteristics are extended to make it have the characteristics of open world. Among them, there are three fault modes in the rotor of the motor, F1 means the rotor is unbalanced, F2 means the rotor is out of alignment, and F3 means that the support is loose. Three vibration acceleration sensors were placed in different installation positions in order to collect vibration signals. The frequency amplitude of acceleration vibration at three different frequencies of Freq1, Freq2 and Freq3 is known as the fault characteristic variable. $\left\{F1,F2,F3\right\}$ incomplete framework for fault identification. After modification of the data in the literature modeling results [41], it is expanded from the closed world to the open world. Table 2 shows the failure data reported by sensors at different frequencies.

### 4.1. Uncertainty Measure of BPAs with ODE

Different sources of information, such as the different sensors in this example, can yield data of different reliability. Therefore, the uncertainty of the mass function of evidence modeled can be measured by the proposed Open Deng entropy. For example, for the evidence modeling results of accelerated vibration frequencies Freq1, Freq2, and Freq3, the uncertainty measurement results are shown in Table 3 according to the entropy measurement formula of the open world described in Equation (16).

As for the uncertainty measurement results that are mentioned in Table 3, for the acceleration vibration frequency Freq1 and evidence $E_{ode}\left(m_{s1}\right)$, the calculation equation is as follows:

(26) $$\begin{array}{cc} E_{ode}\left(m_{s1}\right)= & -0.8176log_{2}\left(\frac{0.8176}{2^{1}-1}e^{\frac{0.0268}{3}}\right)-0.0003log_{2}\left(\frac{0.0003}{2^{1}-1}e^{\frac{0.0268}{3}}\right)\\ & -0.1553log_{2}\left(\frac{0.1553}{2^{1}-1}e^{\frac{0.0268}{3}}\right)=0.8919 \end{array}$$

### 4.2. Mass Function Data Modification Based on ODE

The uncertainty measurement results presented in Table 3 are used for evidence data modification. Using the ODE calculation results as the weighting factor of each sensor report. After normalization, the weights of each group with a small acceleration vibration frequency of Freq1 are calculated, as follows:

(27) $$w_{s1}=E_{ode}\left(m_{s1}\right)/\sum_{i=1}^{3}E_{ode}\left(m_{si}\right)=0.4191,$$

(28) $$w_{s2}=0.3382,w_{s3}=0.2427.$$

Table 4 shows the calculation results of evidence weight under each frequency.

Based on the weight factor in Table 4, it is applied to the following mass functiondata modification formula:

(29) $$m_{w}\left(\cdot \right)=\sum_{i=1}^{3}w_{si}m_{si}.$$

For the example of acceleration vibration frequency Freq1, the modified mass function is calculated:

(30) $$m_{w}\left(\left\{F2\right\}\right)=0.5923,$$

(31) $$m_{w}\left(\left\{F3\right\}\right)=0.0005,$$

(32) $$m_{w}\left(\left\{F1,F2\right\}\right)=0.0904,$$

(33) $$m_{w}\left(\phi \right)=0.3201.$$

The calculated results are shown in Table 5 for the modified mass function under different vibration acceleration frequencies.

### 4.3. Data Fusion Based on Generalized Rules of Evidence Combination

In the open world, since the mass function of the empty set is no longer 0, the classical Dempster combination rule under the hypothesis of the closed world is no longer applicable, so the generalized evidence combination rule [9] is adopted for evidence fusion.

Two generalized evidence combination rules are needed to fuse the revised three groups of the same mass function values. The calculation results of frequency Freq1 are as follows:

(34) $$\begin{array}{cc} \; & m\left(\left\{F2\right\}\right)={\left({\left(m_{w}⊕m_{w}\right)}_{1}⊕m_{w}\right)}_{2}\left(\left\{F2\right\}\right)=0.9909, \end{array}$$

(35) $$\begin{array}{cc} & m\left(\left\{F3\right\}\right)=0,m\left(\left\{F1,F2\right\}\right)=0.0023,m\left(⌀\right)=0.0328. \end{array}$$

**Figure 3:**
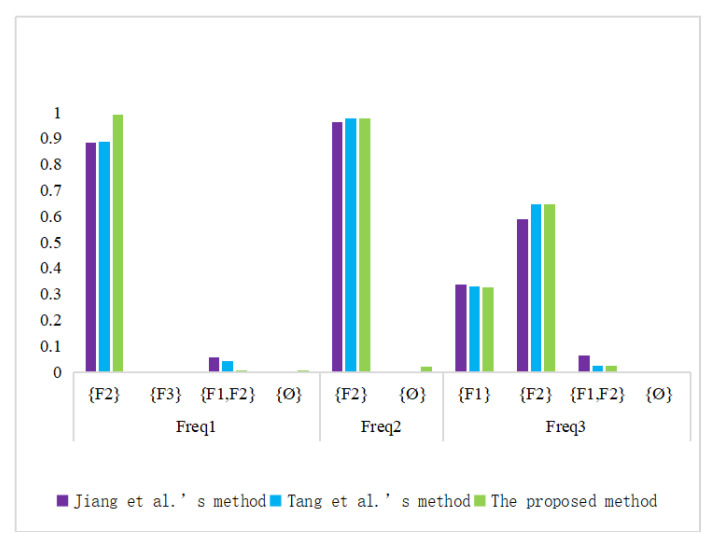
Sensor data fusion results with different methods.

Table 6 and Figure 3 show the fusion results at different frequencies.

The fusion results of Open Deng entropy and generalized evidence combination rule show that F2 has the highest confidence support level under any test frequency condition. Therefore, we can determine the fault type to be F2. Moreover, according to Table 6 and Figure 3, by comparing different methods, we can clearly see that the data fusion results of the proposed method are consistent with those of other literature, which verifies its effectiveness. In addition, the data fusion results of this method have a higher level of confidence support for the fault conclusion, which is more conducive to the application in practical engineering.

## 5. Conclusions

In this paper, an uncertainty measurement method that is based on Deng entropy named ODE is proposed. This method is not only compatible with Deng entropy, but it can effectively quantify the uncertainty of closed and open world. Meanwhile, this method takes into account the sources of uncertain information in the Dempster–Shafer evidence theory framework that are not considered by other existing methods, including the uncertain information brought by the incomplete FOD and the non-zero mass function of empty set. In addition, the proposed method takes into account the sources of uncertain information in the Dempster–Shafer evidence theory framework that are not considered in some existing methods, including the uncertain information brought by the incomplete FOD and the non-zero mass function of empty set. An information fusion method is designed based on the ODE in order to verify the validity and applicability of ODE. Examples and applications verify the rationality and validity of the method. In addition, the limitations and problems of this method are discussed in the open world.

There are still some open issues worthy for further discussion on the ODE. The first problem is that the proposed ODE only considers the mass function of a non-zero empty set as the characteristic factor in the calculation formula, and it does not measure the uncertainty information of the mass function of a non-zero empty set separately. The second problem is that the ODE satisfies the nature of Deng entropy. However, it is also not fully satisfied the characteristics of “set consistency”, “sub-additivity”, and “additivity”. The following work should address more reasonable properties in the open world assumption.

## Figures and Tables

**Table 1:** Uncertainty measurement results of different methods in Example 3.

Uncertainty Measure	$\left|X\right|=2$	$\left|X\right|=3$	$\left|X\right|=5$	$\left|X\right|=7$	$\left|X\right|=9$
$E_{d}\left(m\right)$	-	-	-	-	-
$H\left(m\right)$	1.4855	1.4855	1.4855	1.4855	1.4855
$E_{ode}\left(m\right)$	0.8051	0.8653	0.9133	0.9340	0.9454

**Table 2:** Data for fault diagnosis modeled as BPAs [46].

	Freq1					Freq2			Freq3			
	{F2}	{F3}	{F1,F2}	∅		{F2}	∅		{F1}	{F2}	{F1,F2}	∅
$m_{s_{1}}\left(\cdot \right)$	0.8176	0.0003	0.1553	0.0268		0.6229	0.3771		0.3666	0.4563	0.1185	0.0586
$m_{s_{2}}\left(\cdot \right)$	0.5658	0.0009	0.0646	0.3687		0.7660	0.2341		0.2793	0.4151	0.2652	0.0404
$m_{s_{3}}\left(\cdot \right)$	0.2403	0.0004	0.0141	0.7452		0.8598	0.1402		0.2897	0.4331	0.2470	0.0302

**Table 3:** Measurement results of uncertainty in sensor report based on Open Deng entropy (ODE).

$E_{\mathrm{ode}}\left(\cdot \right)$	Freq1	Freq2	Freq3
$E_{ode}\left(m_{s_{1}}\right)$	0.8919	0.3124	1.5732
$E_{ode}\left(m_{s_{2}}\right)$	0.7198	0.2084	1.9500
$E_{ode}\left(m_{s_{3}}\right)$	0.5166	0.1294	1.9164

**Table 4:** BPAS weighting factor based on ODE after normalization.

$w_{\mathrm{si}}$	Freq1	Freq2	Freq3
$w_{s1}$	0.4191	0.4805	0.2892
$w_{s2}$	0.3382	0.3205	0.3585
$w_{s3}$	0.2427	0.1990	0.3523

**Table 5:** Modified mass function based on ODE.

	Freq1					Freq2			Freq3			
	{F2}	{F3}	{F1,F2}	∅		{F2}	∅		{F1}	{F2}	{F1,F2}	∅
$m_{w}\left(\cdot \right)$	0.5923	0.0005	0.0904	0.3201		0.7159	0.2841		0.3082	0.4334	0.2164	0.0420

**Table 6:** Sensor data fusion results with different methods.

	Freq1					Freq2			Freq3			
	{F2}	{F3}	{F1,F2}	∅		{F2}	∅		{F1}	{F2}	{F1,F2}	∅
Jiang et al.’s method [46]	0.8861	0.0002	0.0582	-		0.9621	-		0.3384	0.5904	0.0651	-
Tang et al.’s method [47]	0.8891	0.0003	0.0427	-		0.9784	-		0.3303	0.6459	0.0238	-
The propose method	0.9909	0.0000	0.0023	0.0328		0.9771	0.0229		0.3285	0.6466	0.0248	0.0001
